# The impact of Facebook use on self-reported eating disorders during the COVID-19 lockdown

**DOI:** 10.1186/s12888-021-03628-x

**Published:** 2021-12-07

**Authors:** Giuseppe Mannino, Laura Salerno, Rubinia Celeste Bonfanti, Gaia Albano, Gianluca Lo Coco

**Affiliations:** 1 LUMSA University, Palermo, Italy; 2 Department of Psychology, Educational Science and Human Movement, University of Palermo, Viale delle Scienze, Edificio 15, 90128 Palermo, Italy

**Keywords:** COVID-19, Quarantine, Disordered eating cognitions, Passive Facebook use, Maladaptive Facebook use

## Abstract

**Background:**

The social isolation due to the COVID-19-related lockdown has had an impact on social media consumption around the world. This study examines the relationship between fear of COVID-19, Facebook use and disordered eating.

**Methods:**

Structural Equation Modeling was used to analyse two-wave survey data (T0: Italian first lockdown; T1: after two months) during the pandemic. Young adults with self-reported dysfunctional eating behaviors (*N* = 115; 91.3% females; mean age = 28.60 ± 7.31) were recruited to complete an online survey at T0; 66 participants (92.4% females; mean age = 28.85 ± 7.85) completed the survey at T1. They were assessed on Facebook use, dysfunctional eating cognitions, and fear of COVID-19.

**Results:**

Participants’ disordered eating cognitions increased during the pandemic. At T0, higher fear of COVID-19 was positively associated to time spent on Facebook, which in turn predicted disordered eating cognitions at T1. Moreover, maladaptive Facebook use mediated the relationship between daily time on Facebook and Shape concerns.

**Conclusions:**

The findings of this study suggest an influence of dysfunctional Facebook use in increasing disordered eating cognitions during the pandemic.

## Background

The coronavirus disease 2019 (COVID-19) is a global pandemic which emerged in Wuhan, Hubei Province of China, at the end of December 2019 and spread to almost all countries across the globe in a few months. COVID-19 has infected individuals worldwide with severe consequences for public health. During the first stages of the pandemic, when vaccine was not yet available, most governments adopted rigid restrictions and lockdown to reduce the spread of COVID-19 across populations and millions of people experienced social distancing or mandatory mass quarantine for several weeks. The Italian government instituted extraordinary public health measures such as shutting down all commercial activities, closing borders and mass quarantine on March 9, 2020, and prolonged these restrictions until the 4th of May. Even later, when the mass quarantine was lifted, a large number of preventive and protective measures aimed at fostering social distancing and limiting the mobility of population, were adopted. At the time of writing, some 104,000 people have died in Italy and more than 3,300,000 confirmed cases have been detected, with the earliest and largest COVID-19 outbreak in Europe. Over the last year, psychosocial research has focused on assessment of Fear of COVID-19, i.e. fear of personal infection or infecting one’s loved ones among people exposed to the infection outbreak [1]. High levels of fear were common in all countries [2–5]; and there is evidence on their association with psychological distress during the pandemic [6, 7]. The experience of fear during COVID-19-related home confinement has had the potential to drive people to staying socially connected by social media [8]. Wiederhold [9] suggested that social media use may have been used during the COVID-19 pandemic as a way to alleviate anxiety, by connecting people with others, creating a sense of normalcy and sharing information with friends and relatives. However, in vulnerable clinical populations such as those with eating disorders, excessive media exposure may increase eating disorder risk and symptoms [10]. The current study examines the link between fear of COVID-19, Facebook use and dysfunctional eating concerns in individuals suffering from disordered eating behaviors during the pandemic.

### COVID-19 and eating disorders

Although quarantine and social distancing are important preventive measures during major infectious disease outbreaks, there is evidence that they may be associated with negative psychological effects [11]. Previous studies reported heightened levels of psychosocial distress after the rise of COVID-19 within the general population [12–14]. Moreover, there is preliminary evidence regarding the impact of restrictions due to COVID-19 on the mental state of people who were suffering from mental health problems before the pandemic, who may be more at risk of increasing distress and worsening of their mental health status due to the disruption of treatment services [15, 16].

More specifically, the few studies which investigated the impact of the COVID-19 outbreak on symptomatic distress in people with eating disorders (EDs) have provided mixed findings. Clinicians and researchers who work with people suffering from EDs and their carers, argued that patients may have to deal with an exacerbation of their eating disorder symptoms as a consequence of the pandemic [17, 18], but more empirical research is needed to further support this negative path. There is initial evidence showing that people with EDs reported worsening on both general (e.g. anxiety, depression, post-traumatic stress symptoms, obsessive-compulsive symptoms, stress, loneliness) and specific (e.g. purging, social insecurity, body dissatisfaction, shape and weight concerns, drive for physical activity) psychopathology during the pandemic [19, 20]. To date, the first meta-analysis that examined the effects of the COVID-19 lockdown on eating disorders [21] showed that the majority of individuals with EDs reported symptomatic worsening during the home-confinement. However, most of previous studies only relied on cross-sectional data [20] or retrospective design [17, 19] and the few research that examined changes in EDs symptoms before and during the lockdown showed mixed findings.

In addition, qualitative studies also suggested a multifaceted experience of people with EDs during the pandemic [15, 22]. It was suggested that the impact of COVID-19 has been experienced not only as a catalyst for disordered eating but also as a trigger to develop a new awareness of individual behavioral intentions and/or coping strategies or a drive for recovery [23–25].

### Facebook use and eating disorders

Among the paths that may lead to an exacerbation of symptomatic distress during the pandemic, the effects of social media play an important role [10]. The negative impact of social media, such as Facebook, on fostering and maintaining both disordered eating behavior and cognitions is well-documented [26–28]. However, prior research regarding the association between amount of time spent on Facebook and disordered eating or body image concerns, resulted in mixed findings. Some studies suggested that time spent on SNSs is related to higher eating disorder symptoms and concerns [26, 29, 30]. One of the few longitudinal studies on this issue showed that more frequent SNS use predicted increased body dissatisfaction among adolescents 18 months later, but body dissatisfaction did not predict SNS use [31]. On the contrary, other studies found that time spent using social media was not a significant predictor of body dissatisfaction, while the number of social media sites visited was [32]. Moreover, it was shown that overall Facebook usage was not significantly correlated with weight dissatisfaction, a drive for slimness, slim-ideal internalization, and self-objectification [29], whereas Walker and colleagues [28] found that college-aged women who engaged in more intense Facebook usage were less likely to report disordered eating behavior. Given the inconsistent evidence for strong connections between the amount of time spent on Facebook and EDs-related symptoms, understanding which negative usage patterns can drive increasing levels of dysfunctional eating concerns and behavior during the COVID-19 pandemic, is a worthwhile research objective.

It is worth noting that different patterns of Facebook use have received research attention in recent literature. Prior research showed that maladaptive Facebook usage (i.e., the tendency to evaluate oneself negatively in comparison to one’s Facebook friends or engage in social comparison) was associated with greater bulimic symptoms and over-eating episodes [33]. On the other hand, passive consumption of social content is the most prevalent social media activity, and it seems to be related to the individual’s distress [34]. Specifically, it refers to a constant monitoring of what one’s social connections (e.g. Facebook friends) do, think and talk about on Facebook without direct exchanges, and passive users are more likely to believe that others are more satisfied with their body [35, 36]. Passive use is comprised of two different domains, i.e. social connection, with a tendency to browse one’s own news feed, reading contents and clicking the “like” button; and social comparison, with a tendency to view friends’ and non-friends’ profiles [37]. Saffran and colleagues [38] showed that looking at Facebook pages without communicating was associated with greater eating disorder psychopathology, according to research which showed that passive SNS usage negatively predicted users’ well-being [39]. It could be hypothesized that a passive use of Facebook can heighten feelings of inadequacy and negative self-evaluation in people suffering from eating problems, due to an upward, online, social comparison, which consists in a comparison with models and artificially high standards (e.g. a high social activity, such as comments and virtual “likes” conveying the person’s popularity and healthy habits).

In summary, the examination of Facebook usage patterns and their associations with problematic eating behavior is an important research topic to pursue in the context of COVID-19-related home confinement, given that people moved to online social media interactions in mass [28] and spent less time communicating face to face. Although some scholars argued a positive side to social media use during COVID-19-related quarantine [40, 41], it is also likely that people suffering from eating problems would be involved more negatively in on-line Facebook communities during the pandemic, given the heightened feelings of loneliness and the lack of ongoing treatment that was being disrupted [24, 42].

### The present study

**Fig. 1:**
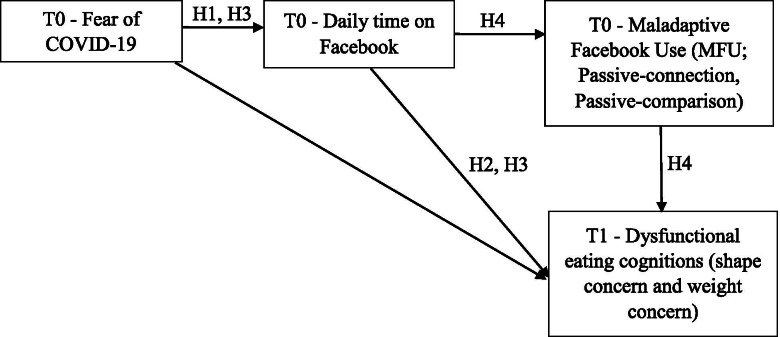
Theoretical model of the study

Although the investigation of the effects of social media use on eating disorder risks and symptoms is a research priority during the pandemic crisis [10], to date, the negative impact of Facebook use on disordered eating behavior and cognitions during the COVID-19 crisis has received limited research attention. In the current investigation, we build upon prior findings which provided support for the negative impact of Facebook use on many detrimental outcomes, including body image concerns and disordered eating, with certain types of activities identified as particularly problematic, such as viewing and uploading photos and seeking negative feedback via status updates [43–45]. Therefore, in this study, we will focus on people suffering from disordered eating behavior with the aim of extending previous research on the association between social media use and disordered eating during the first Italian lockdown (T0) and the following period when some restrictions were eased (T1, after 2 months). For the purposes of the study, we recruited the members of a Facebook online community with people suffering from dysfunctional eating behavior, who were highly engaged in Facebook activities during the COVID-19 pandemic. Specifically, the following hypotheses will be tested (see Fig. 1 for the theoretical model):

- H1. Higher Fear of COVID-19 would be associated with higher daily Time spent on Facebook during the lockdown (T0).
- H2. Higher daily Time spent on Facebook at T0 would predict greater disordered eating cognitions (i.e. Shape Concern – H2a, and Weight Concern – H2b) at T1.
- H3. Daily time spent on Facebook would mediate the relationship between Fear of COVID-19 and dysfunctional eating cognitions (i.e. Shape Concern – H3a, and Weight Concern – H3b) at T1.
- H4. Dysfunctional Facebook use (i.e. maladaptive Facebook use – H4a, passive use-social connection – H4b, and passive use-social comparison – H4c) at T0 would mediate relations between daily Time spent on Facebook at T0 and dysfunctional eating cognitions at T1.
- H5. ED symptoms would exacerbate from T0 to T1 during the COVID-19 lockdown.

Finally, two covariates were controlled in the analyses. Specifically, we added disordered eating cognitions at T0 (i.e. EDE-Q Shape Concern and EDE-Q Weight concern) as control variables.

## Method

### Participants

Participants were recruited in a Facebook online community focused on Eating Disorders, whose members suffer from dysfunctional eating behavior. The study included two waves (see Procedure section for details). One hundred and twenty participants (10 males and 110 females; mean age = 28.58, SD = 7.19; age range = 19–50) consented to participate and completed an online survey at T0. Of these, 71 participants (5 males and 66 females; mean age = 28.80, SD = 7.63; age range = 19–49) completed the second survey at T1 (response rate 59.2%). Five participants were identified as outliers and subsequently excluded from the analyses (see the Plan of Data Analysis section), thus 115 participants and 66 participants were considered for the two waves, respectively. Demographic information as well as data about changing life conditions and impacts resulting from COVID-19 pandemic are reported in Table 1. Of the 115 participants at T0, nineteen (16.5%) were underweight (BMI ≤18.5 Kg/m^2^), seventy-five participants (65.2%) were normal weight (18.5 ≤ BMI ≤ 24.9 Kg/m^2^), and fifteen participants (13.1%) were overweight or obese (BMI ≥ 25.0 Kg/m^2^). For six participants (5.2%) data on BMI were missing. According to the EDE-Q cut-off for different BMI range [46], 55.0% of participants with available data on BMI falling within the clinical range (16 participants for the underweight subgroup, 34 for the normal weight subgroup and 10 for the overweight/obese subgroup).

**Table 1:** Demographics and information about changing life conditions and impact resulting from COVID-19 pandemic

	Participants T0 (N = 115)	Participants T1 (*N* = 66)
Age, M (SD)	28.60 (7.31)	28.85 (7.85)
Gender, N (%) females	105 (91.3)	61 (92.4)
Educational level, N (%)		
*8 years of education*	6 (5.2)	4 (6.1)
*13 years of education*	60 (52.2)	32 (48.5)
*Degree or post-degree*	49 (42.6)	30 (45.4)
Marital status, N (%)		
*Married/in a relationship*	59 (51.3)	35 (53.0)
*Single/separated-divorced/widowed*	56 (48.7)	31 (47.0)
Exits from place of residence^a^, N (%) yes	76 (66.1)	–
Number of exits from place of residence, M (SD)	2.82 (3.62)	–
With whom the quarantine was spent, N (%)		
*Alone*	10 (8.7)	–
*With other people (one or more)*	101 (87.8)	–
*missing*	4 (3.5)	–
Direct contact with someone positive to Covid-19, N (%) yes	14 (12.2)	–
Perceived risk, for parents/close family members, of contracting COVID-19, N (%)		
*Very low or low risk*	21 (18.3)	–
*Medium risk*	38 (33.0)	–
*High or very high risk*	52 (45.2)	–
*missing*	4 (3.5)	–

Note: ^a^ = for grocery shopping and/or work and/or basic needs, over the preceding week. For this table, “Perceived risk, for parents/close family members, of contracting COVID-19” was calculated as a mean of responses about “risk perceived for parents” and “risk perceived for close family members”; scores range from 0 to 10 and are categorized as very low or low (points from 1 to 4), medium (points 5 and 6), high or very high (points from 7 to 10)

### Measures

At T0, the first part of the questionnaire was used to collect information about participants’ characteristics, including age, gender, education, marital status and hours of daily Facebook usage. In the next part, some questions regarding changing life conditions and impact resulting from the pandemic (i.e. information about Quarantine condition) have been inserted. Finally, data about fear of COVID-19 pandemic, passive Facebook use, maladaptive Facebook use and eating disorder symptoms were collected. At T2, data about eating disorder symptoms were collected.

#### Information about quarantine condition (T0)

Participants were asked to answer ten questions regarding changing life conditions and impact resulting from the COVID-19 pandemic, based on the items in the review by Brooks et al. [11]. Specifically, the questions explored the following: 1) the possibility of going out during the last week for specific reasons (2 questions), 2) companionship during the quarantine (1 question), 3) direct contact with someone positive for COVID-19 (1 question), 4) perceived risk of contracting the virus (2 questions). Responses are rated on a ten-point scale (from 0 to 10).

#### Fear of COVID-19 pandemic (T0)

Fear of COVID-19 pandemic was assessed with the Fear of COVID-19 Scale (FC-19S) [1]. The FC-19S is a 7-item unidimensional measure (e.g. “*When watching news and stories about Coronavirus-19 on social media, I become nervous or anxious*”) which showed robust psychometric properties in previous studies [1, 5] as well as in the present study (Cronbach’s α = .88). Items were rated on a 5-point Likert scale, from 1 (*Strongly Disagree*) to 5 (*Strongly Agree*) with higher overall scores indicating more severe fear of COVID-19 pandemic.

#### Maladaptive Facebook questionnaire (T0)

Three items belonging to *Maladaptive Facebook Usage Scale* (MFU) [33] were used to assess whether people use Facebook in a maladaptive way. MFU is a 7-item questionnaire (e.g. “*I tend to read the status updates of others to see if they are feeling the way I am*”). Items were rated on a 7-point Likert scale, from 1 (*Strongly Disagree*) to 7 (*Strongly Agree*) with higher scores indicating greater tendencies to seek online social comparison and negative evaluation. The MFU showed acceptable internal consistency (Cronbach’s α = .66; mean inter-item correlation: .41).

#### The passive use of Facebook (T0)

Type of Facebook use was assessed with the 17-item *Active and Passive Facebook Use Scale* (APUF) [37], which measures active and passive Facebook usage. For the purposes of this study, only the two passive Facebook use subscales were considered, specifically passive use social connection (4 items) (e.g. *“Reading posts”*) and passive use social comparison (3 items) (e.g. “*Viewing friends’ profile*”). Answers included a 7-point scale (from 1 = *Never* to 7 = *More than once a day*). The two passive Facebook Use subscales showed acceptable internal consistency (Cronbach’s α = .64 and .76 for passive use social connection and passive use social comparison subscale, respectively; mean inter-item correlation was .32 for passive use social connection subscale).

#### Eating disorders (T0 – T1)

The *Eating Disorder Examination Questionnaire* (EDE-Q 6.0) [47] was used to measure core eating disorder behaviour over the preceding 28 days. The EDE-Q is a self-report questionnaire and comprises 28 items, of which 22 items addressed attitudinal aspects of eating disorder psychopathology and 6 items (which do not contribute to subscale and global scores) evaluated the number of episodes of ED behaviour occurring during the previous 4 weeks. The EDE-Q yields a global score and four subscales: Restraint (5 items; e.g. “Have you tried to exclude from your diet any foods that you like in order to influence your shape or weight (whether or not you have succeeded)?”), Eating Concern (5 items; e.g. “Have you had a definite fear of losing control over eating?”), Shape Concern (8 items; e.g. “Have you had a definite desire to have a totally flat stomach?”) and Weight Concern (5 items; e.g. “Has your weight influenced how you think about (judge) yourself as a person?”). Higher scores indicate greater levels of eating disorder. In this study, both the global and subscale scores showed a good internal consistency (see Table 4).

### Procedure

Respondents for this study were recruited through an online advertisement published in an online Facebook community on eating disorders. All respondents were living in Italy. The first administration (T0) took place from 15th of March to 11th of April 2020 (during the first lockdown in Italy). The second administration (T1) took place from 5th of June to 16th June 2020, during a time of eased restrictions. Participation in the study was on a voluntary basis. The questionnaire was anonymous, but the respondents could indicate their willingness to know the results and provide their contact details. The online questionnaire took approximately 15–20 min to complete. The study was conducted in accordance with the ethical standards of the Italian Psychological Association (AIP), as well as the Declaration of Helsinki. All participants completed statements of informed consent to participate in the study.

### Plan of data analysis

Data were analysed using SPSS, version 22. Attrition analysis was conducted to compare participants who participated versus not-participated in the study at T1. No significant differences on demographics (i.e. age, gender, educational level, and marital status) or ED symptoms at T0 were found. Moreover, Little’s [48] MCAR test was not significant, χ^2^(15) = 5.622, *p* = .985, indicating that drop-out likely occurred at random. Hence, the missing data were dealt with through the expectation maximization [49] algorithm for analyses. Preliminary analyses were conducted to verify the normality of the data distribution [50]. A positive skewed distribution for Fear of COVID-19, APUF-passive use social connection subscale and daily Time of Facebook Use was found. All other variables revealed no substantial violation of normality regarding data distribution (∣Skewness∣ < 1). Five participants identified as outliers were removed to improve the normality of the Fear of COVID-19 scale and the APUF-passive use social connection subscale and square root transformation was used to improve the normality of daily Time of Facebook use. Analyses of transformed data were reported. However, analyses run with or without transformed variables led to similar results. Cronbach alpha (α) was computed for all scales to assess internal consistency and the mean inter-item correlation was also examined for the APUF-passive use social connection and for the MFU scales. Mean inter-item correlations between .15 and .50 indicate adequate internal consistency [51]. Descriptive statistics (mean, standard deviations and percentages) were computed for demographics and variables of interest. Bivariate correlation coefficients between variables were examined. The first four hypotheses of the study (H1, H2, H3 and H4) were tested using Structural Equation Modeling (SEM). The theoretical model tested in this study is displayed in Fig. 1. Given the central role of shape and weight concerns in the relationship with SNS use [26] only EDE-Q shape and weight concern subscales were used for the following analyses. Model testing was performed using Mplus software (version 6.12). The overall goodness of model-fit was assessed using the χ^2^ test statistics (χ^2^/df ratios < 3 indicate reasonable fitting models), the comparative fit index (CFI; values higher than .95 are considered as indicators of good fit) [52], and the root-mean-square error of approximation (RMSEA; values lower than .08 indicate good fit) [53, 54]. Finally, 95% confidence intervals (CIs) were computed using 5000 bootstrap resamples for indirect effects [55]. CIs that do not contain a zero value indicate a significant indirect effect. Differences between T0 and T1 (H5) on EDs symptoms were evaluated by paired sample t-test.

## Results

### Cross-sectional findings: the relationship between Fear of COVID-19 and daily Time spent on Facebook (H1)

Correlational analyses showed that Fear of COVID was associated with higher daily time spent on Facebook and maladaptive Facebook use during the lockdown (T0). Moreover, passive Facebook use- social comparison was associated to Maladaptive Facebook use at T0 (see Table 2).

**Table 2:** Means, standard deviation and correlations between variables

	M	SD	Skewness	Kurtosis	1	2	3	4	5	6	7	8
1. T0 - Fear of COVID-19	14.79	5.92	.982	.787								
2. T0 - Daily Time on Facebook	3.39	1.99	.562	−.145	.325**							
3. T0 - Maladaptive Facebook Use (MFU)	6.55	3.94	.966	−.167	.267**	.322**						
4. T0 - Passive use - social connection (APUF)	23.87	3.90	−.964	.112	.155	.359**	.153					
5. T0 - Passive use - social comparison (APUF)	8.99	4.34	.671	−.140	.108	.168	.399**	.412**				
6. T0 - Shape Concern (EDE-Q)	3.76	1.98	−.450	−1.211	.228*	.080	.295**	.053	.169			
7. T0 - Weight Concern (EDE-Q)	3.22	1.99	−.168	−1.358	.221*	.085	.317**	.036	.175	.953**		
8. T1 - Shape Concern (EDE-Q)	4.13	1.99	−.355	−1.276	.246**	.181	.425**	.070	.245**	.951**	.924**	
9. T1 - Weight Concern (EDE-Q)	3.72	1.93	−.088	−1.238	.205*	.165	.405**	.029	.258**	.890**	.914**	.960**

Note: T0 = total Italian lockdown - from March 15, 2020 to April 11, 2020; T1 = following period of time in which, even if the mass quarantine was lifted, a large number of preventive and protective measures were adopted - from June 5, 2020 to June 16, 2020; Daily time on Facebook = hours of daily use of Facebook; MFU = Maladaptive Facebook Use; APUF = Active and Passive Facebook Use Scale; EDE-Q = Eating Disorder Examination Questionnaire. * *p* < .05, ** *p* < .01

**Fig. 2:**
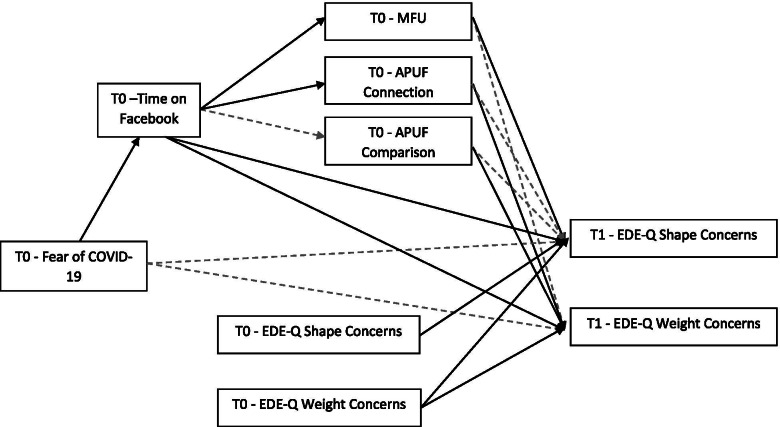
Structural equation model. Errors were omitted from the diagram. Non-significant parameters are represented by dashed grey lines. The residuals of MFU and APUF comparison (p < .01), the residuals of APUF connection and APUF comparison (p < .001), as well as T1 EDE-Q Shape and Weight concerns (p < .001) are significantly and positively correlated in the model; however, for clarity purposes, they are not showed in the figure

In order to examine the study hypotheses, we tested the theoretical model depicted in Fig. 1 by SEM. The results of SEM approach are displayed in Table 3. All fit indices suggested that the model (see Fig. 2) provided only a modest fit to the data (χ^2^ = 78.538; df = 16, χ^2^ /df = 4.91; CFI = .914; RMSEA = .184; RMSEA 90% C.I. = .145–.226), consequently, the modification indices were used to improve the fit. More specifically, the model was modified by adding two covariances (i.e. between MFU and APUF passive use-social comparison and between APUF passive use-social comparison and APUF passive use-social connection) and a relationship between EDE-Q Weight concern at T0 and EDE-Q Shape concern at T1. These modifications improved the model fit (χ^2^ = 19.541; df = 13; χ^2^/df = 1.50; CFI = .991; RMSEA = .066; RMSEA 90% C.I. = .000–.123). The model accounts for 93% of the variance in T1-EDE-Q Shape concern (R^2^ = .927), and for 85% of the variance in T1-EDE-Q Weight concern (R^2^ = .852). Consistently with the first hypothesis of the study (H1), Fear of COVID-19 was positively associated to daily time spent on Facebook at T0.

**Table 3:** SEM analysis

	*b (β)*	*SE*	*t*	*p*
*Direct paths*				
T0 Fear of Covid→T0 Time on Facebook	.028 (.326)	.008	3.698	.000
T0 Time on Facebook → T0 MFU	2.460 (.322)	.687	3.580	.000
T0 Time on Facebook →T0 APUF Connection	2.715 (.359)	.650	4.179	.000
T0 Time on Facebook →T0 APUF Comparison	1.410 (.168)	.875	1.611	.107
T0 MFU → T1 EDE-Q Shape concern	.059 (.122)	.017	3.546	.000
T0 MFU → T1 EDE-Q Weight concern	.041 (.087)	.025	1.637	.102
T0 APUF Connection →T1 EDE-Q Shape concern	−.019 (−.039)	.015	−1.256	.209
T0 APUF Connection →T1 EDE-Q Weight concern	−.039 (−.082)	.019	−2.089	.037
T0 APUF Comparison→T1 EDE-Q Shape concern	.021 (.048)	.012	1.799	.072
T0 APUF Comparison→T1 EDE-Q Weight concern	.042 (.096)	.019	2.173	.030
T0 Time on Facebook →T1 EDE-Q Shape concern	.309 (.084)	.102	3.017	.003
T0 Time on Facebook →T1 EDE-Q Weight concern	.333 (.092)	.135	2.468	.014
T0 Fear of Covid→ T1EDE-Q Shape concern	−.005 (−.016)	.009	−.542	.588
T0 Fear of Covid→ T1 EDE-Q Weight concern	−.012 (−.039)	.014	−.861	.389
*Control variables*				
T0 EDE-Q Shape concern→ T1 EDE-Q Shape concern	.652 (.677)	.052	12.633	.000
T0 EDE-Q Weight concern→ T1 EDE-Q Weight concern	.847 (.906)	.042	20.335	.000
T0 EDE-Q Weight concern→ T1 EDE-Q Shape concern	.261 (.273)	.057	4.544	.000
*Indirect effects*				
T0 Fear of Covid→T0 Time on Facebook → T1 EDE-Q Shape concern	.009 (.027)	.004	2.292	.022
T0 Fear of Covid→T0 Time on Facebook → T0 MFU → T1 EDE-Q Shape concern	.004 (.013)	.002	1.934	.053
T0 Fear of Covid→T0 Time on Facebook → T0 P-connection →T1 EDE-Q Shape concern	−.001 (−.005)	.001	−1.054	.292
T0 Fear of Covid→T0 Time on Facebook →T0 P-comparison →T1 EDE-Q Shape concern	.001 (.003)	.001	1.054	.292
T0 Fear of Covid→T0 Time on Facebook → T1 EDE-Q Weight concern	.009 (.030)	.005	1.975	.048
T0 Fear of Covid→T0 Time on Facebook → T0 MFU → T1 EDE-Q Weight concern	.003 (.009)	.002	1.234	.217
T0 Fear of Covid→T0 Time on Facebook →T0 P-connection → T1 EDE-Q Weight concern	−.003 (−.010)	.002	−1.468	.142
T0 Fear of Covid→T0 Time on Facebook →T0 P-comparison → T1 EDE-Q Weight concern	.002 (.005)	.002	1.089	.276
T0 Time on Facebook → T0 MFU → T1 EDE-Q Shape Concern	.145 (.039)	.058	2.491	.013
T0 Time on Facebook →T0 P-connection →T1 EDE-Q Shape concern	−.051 (−.014)	.045	−1.136	.256
T0 Time on Facebook → T0 P-comparison →T1 EDE-Q Shape concern	0.30 (.008)	.027	1.124	.261
T0 Time on Facebook → T0 MFU → T1 EDE-Q Weight concern	.101 (.028)	.074	1.371	.170
T0 Time on Facebook →T0 P-connection → T1 EDE-Q Weight concern	−.107 (−.030)	.065	−1.633	.102
T0 Time on Facebook →T0 P-comparison → T1 EDE-Q Weight concern	.059 (.016)	.049	1.195	.232

Note: *APUF* Active and Passive Facebook Use Scale, *connection* Passive use-social connection, *comparison* Passive use-social comparison, *MFU* Maladaptive Facebook Use, *EDE-Q* Eating Disorder Examination Questionnaire

### Longitudinal findings: relationship between fear of COVID-19, Facebook use and EDs (H2, H3, H4) and differences on EDs symptoms between T0 and T1 (H5)

Consistently with the second hypothesis of the study, standardized parameter estimates indicate that higher daily time spent on Facebook predicted higher Shape concern (H2a) and Weight concern (H2b) at T1. Also, consistently with H3a and H3b, daily time spent on Facebook mediates the relations between fear of COVID-19 and dysfunctional eating cognitions (for Shape concern = standardized indirect effect value = .027, *p* < .05, 95% CI .008–.047; for Weight concern = standardized indirect effect value = .030, p < .05, 95% CI .005–.055). Furthermore, a totally mediated model without direct effects of fear of COVID-19 on disordered eating cognitions, was estimated. Then the fit of this second model was compared to the first model by using the difference between the two model chi-squares [56]. The totally mediated model had a good fit (χ^2^ = 20.691, df = 15, χ^2^ /df = 1.38, CFI = .992, RMSEA = .057, RMSEA 90% CI = .000–.112). The paths from fear of COVID-19 to daily time on Facebook (β = .326, *p* < .001), as well as the paths from daily Time on Facebook to EDE-Q Shape concern (β = .080, *p* < .01) and EDE-Q Weight concern (β = .082, *p* < .05) remained significant. Also the indirect effects of fear of COVID-19 on EDE-Q subscales remained significant (for Shape concern = standardized indirect effect value = .026, p < .05, 95% CI = .009–.043; for Weight concern = standardized indirect effect value = .027, p < .05, 95% CI = .005–.049). Moreover, the chi-square difference (Δχ^2^ = 1.15, Δdf = 2) favoured the model in which the relationship between fear of COVID-19 and disordered eating cognitions is fully mediated by daily time on Facebook.

The mediational role of maladaptive Facebook use in the relationship between daily time on Facebook and disordered eating cognition (H4) was only partially confirmed. More specifically, regarding maladaptive Facebook use (H4a), standardized parameter estimates indicate that higher daily time spent on Facebook was associated with greater maladaptive Facebook Use, which in turn was related to higher EDE-Q Shape concern at T1, but not to EDE-Q Weight concern. Maladaptive Facebook use mediates the relationship between daily time on Facebook and EDE-Q Shape concern at T1 (*p* < .05, 95% C.I. = .013–.066). Regarding APUF passive use-social connection (H4b), higher daily time on Facebook was related to higher passive use-social connection, which in turn was related to greater Weight Concerns at T1, but the mediational role of APUF passive use-social connection was not significant. Finally, also the mediational role of APUF passive use-social comparison in the relationship between daily time on Facebook and dysfunctional eating cognitions (H4c) was not significant.

Finally, consistently with the fifth hypothesis of the study (H5), at T1 participants showed higher levels of eating disorder symptoms (EDE-Q total score). More specifically, levels of eating concerns, shape concerns and weight concerns increased during the first months of COVID-19 pandemic (Table 4). No significant difference was found on the EDE-Q restraint subscale.

**Table 4:** Changes in eating disorder symptoms between T0 and T1for EDE-Q global and subscale scores

	T0			T1			t (114)	p	Cohen’s d ES
	M	SD	α	M	SD	α			
EDE-Q - Restraint	2.72	2.05	.89	2.71	1.81	.87	.147	.883	.137
EDE-Q – Eating Concerns	2.23	1.93	.88	2.70	1.91	.90	−6.374	.000	−.594
EDE-Q – Shape Concerns	3.76	1.98	.95	4.13	1.99	.96	−6.406	.000	−.597
EDE-Q – Weight Concerns	3.22	1.99	.87	3.72	1.93	.91	−6.645	.000	−.620
EDE-Q – Total score	2.98	1.87	.97	3.31	1.79	.97	−5.876	.000	−.548

Note: T0 = total Italian lockdown - from March 15, 2020 to April 11, 2020; T1 = following period of time in which, even if the mass quarantine was lifted, a large number of preventive and protective measures were adopted - from June 5, 2020 to June 16, 2020; EDE-Q = Eating Disorder Examination Questionnaire

## Discussion

Social media such Facebook can have a crucial role in disseminating health information and tackling infodemics and misinformation during the COVID-19 pandemic [57]. However, people with disordered eating behaviour may report a maladaptive Facebook use which can negatively impact on their symptomatic behavior. The current study examined the relationships between fear of COVID-19, Facebook use and disordered eating cognitions in the context of the COVID-19-related quarantine.

The current study is the first to examine the role of fear of COVID and dysfunctional Facebook use on eating behavior concerns during the pandemic. Although prior research evidenced a negative impact of Facebook use on fostering and maintaining disordered eating cognitions [27, 28], there is a dearth of studies exploring the link between fear of COVID and social media use during the pandemic. Consistently with the first hypothesis of the study, a direct positive relationship between high fear of COVID-19 and daily time spent on Facebook was found. Thus, increased amounts of time on Facebook may occur as a result of heightened fear of COVID, as a strategy to reduce the negative impact of threats. In the current study we included people belonging to an online Facebook community on eating disorders aimed at offering social support, health advice and recovery-oriented guidance. It is likely that online social support was important for people with a high level of fear of COVID who shared several information about the pandemic [23]. Our findings are in line with previous evidence indicating that daily time spent online with social media activities increased during the COVID-19 crisis [9, 57] and that problematic and excessive social media use is associated with fear of COVID [58]. A previous study on the relationship between COVID-19 anxiety and problematic smartphone use (PSU) [59] hypothesized that the increased PSU during the COVID-19 pandemic could be due to other worries (e.g. social and intimate relationship maintenance, fear of missing out on rewarding experiences) and general anxiety instead to a COVID-19-specific anxiety. In a similar way, the role of moderating variables in the relationship between COVID-19-specific fears and anxiety and the amount of Facebook use may be evaluated in future studies.

Of note, the results of the current study showed a direct positive relationship between daily time on Facebook and eating concerns, consistently with our second hypothesis. This finding seems to be consistent with previous studies which assessed the association between frequency of social media use and eating concerns [26, 60, 61]. Moreover, previous research suggested that higher levels of social media use can be associated with worse mental health symptoms during the pandemic [62]. Although social media such as Facebook can have a crucial role in people’s perception of disease exposure and resultant decision making, individuals with dysfunctional eating behaviour may be more likely to report maladaptive social media use. For instance, people reporting higher levels of social media use could be more likely to be exposed to influential visual material which may promote the slimness ideal and a frequent appearance comparison that present risks for the development or exacerbation of eating concerns [29, 43]. The current study adds to previous literature the role of fear of COVID-19, which can exacerbate ED symptoms through the mediating role of the amount of time spent on Facebook (third hypothesis of the study).

Most importantly, the results of the present study showed a direct link between higher daily time on Facebook and greater maladaptive Facebook use, which in turn predicted greater shape concerns for the individual. The path analysis and bootstrapping results have confirmed the mediating role of maladaptive Facebook use in the relationship between time spent on Facebook and an individual’s shape concerns. Prior research suggested that maladaptive Facebook use (i.e. the tendency to evaluate oneself negatively in comparison to one’s Facebook friends or engage in social comparison) can negatively trigger eating symptoms [33], and our findings add that this increased risk is also likely to occur during the COVID-19 pandemic. Maladaptive social media use has been identified as a potential mental health problem [63], and there is initial evidence showing the association between the frequency of comparing one’s own physical appearance to that of people followed on social media, and body dissatisfaction [64].

However, although the Passive Facebook-social comparison was associated with weight and shape concerns at T1, the negative role of passive Facebook use as a mediational variable in the relationship between time on Facebook and disordered eating cognitions was not supported. Prior research has suggested that passive Facebook use can predict the frequency of making upward social comparisons and consequently it has the capacity to trigger a wide range of emotions such as envy and resentment [65], which in turn may promote the development or exacerbation of eating concerns. A possible explanation for our finding is that the experience of the COVID-19-related lockdown encouraged individuals with eating problems to a greater active than passive Facebook use. It is worth noting that although scholars have extensively examined the association between Facebook use and dysfunctional eating behavior, the specific negative consequences of its active or passive usage needs further investigation [66].

Finally, consistently with our fifth hypothesis, the results of the study showed an exacerbation of ED symptoms for people with dysfunctional eating behaviour (eating concern, shape concern and weight concern, as well as a total score of the EDE-Q) during the COVID-19 pandemic. This finding seems consistent with the preliminary evidence suggesting a worsening of ED psychopathology after the COVID-19 outbreak [17, 18, 25, 67–70]. To date, only a few studies [17, 19, 68] have evaluated, with a retrospective design, changes in ED-specific symptoms at different phases of pandemic management, which are characterized by restrictions and preventive measures with different levels of isolation and limitations to one’s social life. However, in their study, Monteleone and colleagues [19] found opposite results in that social insecurity, body dissatisfaction, binge eating and physical activity were higher during the period of time with higher social restrictions (total lockdown) than the one with a partial “re-opening”. These differences might be due to the different methodologies (i.e. retrospective vs prospective design) as well as to different participants’ characteristics (i.e. ED outpatients vs ED community sample). Thus, further longitudinal studies are needed in order to understand the long-term effects of the COVID-19 pandemic on ED symptoms and the relationship between specific social preventive measures and the trajectory of ED symptoms over time. Castellini and colleagues [68] showed that the COVID-19 pandemic has significantly impacted on ED patients’ symptomatology during the lockdown, when compared to the previous time. Specifically, the pandemic interfered with treatment outcomes for people who suffer from EDs, and remitted patients showed re-exacerbation of binge eating caused by lockdown. Also Fernandez-Aranda and colleagues [17], in a retrospective study, showed an increase in eating symptomatology and in psychopathology of patients with an ED due to home-confinement. These results suggest that the worsening of symptomatology may have already occurred prior to our T0, and our results detected the evolution of the worsening in ED symptoms during and after the home-confinement. The current study makes a contribution to the literature concerning the relationship between Facebook use and psychological outcomes for individuals suffering from dysfunctional eating behavior, and tries to explain the underlying mechanisms that may account for these associations during the COVID-19 pandemic. These findings suggest channelling future research in the direction of these relationships, because underlying mechanisms may be important in informing intervention targets and reducing the negative consequences of using social networks on the part of individuals suffering from dysfunctional eating behavior.

Several limitations should be considered when interpreting the current results. First, we only assessed people from whom we had not received an official diagnosis, but who declared publicly and independently in the Facebook group that they had an eating disorder. Second, all data were only collected from a few online groups on eating disorders and only on Facebook. So, our sample is not a national, representative sample, and this weakens the generalizability of the present findings for future studies. Third, all data were self-reported and might be susceptible to, and affected by response tendencies such as social desirability. Fourth, the sample is unbalanced about the gender of participants (91.3 and 92.4% females at T1 and T2, respectively) and further research is needed to shed some light on gender differences that may influence the relationships between the examined variables. Finally, the response rate of T1 was 57% of T0 and non-respondents might have obtained different results from actual respondents.

## Conclusion

This is the first research that has investigated the impact of dysfunctional Facebook use during the COVID-19 in a sample of individuals suffering from self-reported dysfunctional eating behavior. In summary, a consistent association between social media use and eating concerns was found for these individuals during COVID-19 Italian lockdown. Taken together, these results suggest that during the pandemic, people with dysfunctional eating behaviour may be at risk of a deterioration of ED symptoms and that maladaptive Facebook use may play a role in a negative outcome.

## Data Availability

The dataset generated for this study are available on request from the corresponding author.

## Abbreviations

EDs: Eating Disorders
MFU: Maladaptive Facebook Usage Scale
EDE-Q: Eating Disorder Examination Questionnaire
APUF: Active and Passive Facebook Use
AIP: Italian Psychological Association
PSU: Problematic Smartphone Use
